# Raising the standards of patient‐centered outcomes research in myelodysplastic syndromes: Clinical utility and validation of the subscales of the QUALMS from the MDS‐RIGHT project

**DOI:** 10.1002/cam4.5487

**Published:** 2022-12-19

**Authors:** Fabio Efficace, Karin Koinig, Francesco Cottone, David Bowen, Moshe Mittelman, Kathrin Sommer, Saskia Langemeijer, Dominic Culligan, Kalman Filanovsky, Michael Storck, Alexandra Smith, Corine van Marrewijk, Martin Dugas, Igor Stojkov, Uwe Siebert, Theo de Witte, Reinhard Stauder

**Affiliations:** ^1^ Italian Group for Adult Hematologic Diseases (GIMEMA) Data Center and Health Outcomes Research Unit Rome Italy; ^2^ Department of Internal Medicine V (Hematology and Oncology) Medical University Innsbruck Innsbruck Austria; ^3^ St. James's Institute of Oncology, Leeds Teaching Hospitals Leeds UK; ^4^ Department of Hematology Tel Aviv Sourasky (Ichilov) Medical Center, Sackler Faculty of Medicine, Tel Aviv University Tel Aviv‐Yafo Israel; ^5^ Department of Hematology Radboud University Medical Center Nijmegen The Netherlands; ^6^ Department of Haematology Aberdeen Royal Infirmary Aberdeen UK; ^7^ Department of Medicine Kaplan Medical Center Rehovot Israel; ^8^ Institute of Medical Informatics, University of Münster Münster Germany; ^9^ Epidemiology and Cancer Statistics Group, Department of Health Sciences University of York York UK; ^10^ Institute of Medical Informatics, Heidelberg University Hospital Heidelberg Germany; ^11^ Department of Public Health, Health Services Research and Health Technology Assessment Institute of Public Health, Medical Decision Making and Health Technology Assessment, UMIT ‐ University for Health Sciences, Medical Informatics and Technology Hall in Tirol Austria; ^12^ Departments of Epidemiology and Health Policy & Management Center for Health Decision Science, Harvard Chan School of Public Health Boston Massachusetts USA; ^13^ Institute for Technology Assessment and Department of Radiology Massachusetts General Hospital, Harvard Medical School Boston Massachusetts USA; ^14^ Department of Tumor Immunology Nijmegen Center for Molecular Life Sciences, Radboud University Medical Center Nijmegen The Netherlands

**Keywords:** myelodysplasia, myelodysplastic syndromes, patient‐reported outcomes, quality of life, questionnaire, symptom burden

## Abstract

**Background:**

Clinical decision‐making for patients with myelodysplastic syndromes (MDS) is challenging, and both disease and treatment effects heavily impact health‐related quality of life (HRQoL) of these patients. Therefore, disease‐specific HRQoL measures can be critical to harness the patient voice in MDS research.

**Methods:**

We report a prospective international validation study of the Quality of Life in Myelodysplasia Scale (QUALMS) with a main focus on providing information on the psychometric characteristics of its three subscales: physical burden (QUALMS‐P), emotional burden (QUALMS‐E), and benefit finding (QUALMS‐BF). The analysis is based on patients enrolled from three European countries and Israel, participating to the MDS‐RIGHT Project. The scale structure and psychometric properties of the QUALMS were assessed.

**Results:**

Overall, 270 patients with a median age of 74 years were analyzed and the majority of them (60.3%) had a low MDS‐Comorbidity Index score. Results of the confirmatory factor analysis supported the underlying scale structure of the QUALMS, which, in addition to a total score, includes three subscales: QUALMS‐P, QUALMS‐E, and the QUALMS‐BF. The QUALMS‐P exhibited the highest Cronbach's alpha coefficients. Discriminant validity analysis indicated good results with the QUALMS‐P and QUALMS‐E distinguishing between patients with different performance status, comorbidity, anemia, and transfusion dependency status. No floor and ceiling effects were observed. Responsiveness to change analysis supported the validity of the measure. Patients with a hemoglobin (Hb) level of <11 g/dL at study entry, who subsequently showed an improvement in their Hb levels, also reported a mean score change of 9 and 8 points (scales ranging between 0 and 100) in the expected direction of the QUALMS‐E and QUALMS‐P, respectively.

**Conclusions:**

Our study provides additional validation data on the QUALMS from the international MDS‐RIGHT Project. The use of this disease‐specific HRQoL measure may contribute to raise quality standards of patient‐centered outcomes research in MDS.

## Graphical abstract

The QUALMS is a Patient‐Reported Outcome measure to assess health‐related quality of life in patients with myelodysplastic syndromes. This study, conducted across three European countries and Israel, supports the validity of the QUALMS and provides novel information on the psychometric performance of its three subscales (QUALMS‐P; QUALMS‐E; QUALMS‐BF).

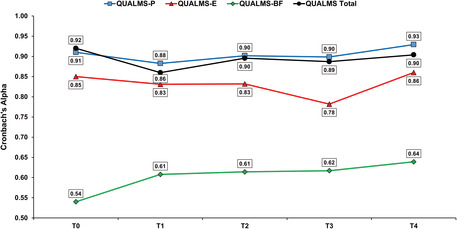

## 1 INTRODUCTION

Clinical decision‐making for patients with myelodysplastic syndromes (MDS) is challenging due to considerable heterogeneity of disease biology and concomitant health conditions at the time of clinical presentation.^1^, ^2^

Patients with MDS typically report a number of troublesome symptoms, which compromise their daily activities and health‐related quality of life (HRQoL)^3^ and often lead to high levels of distress.^4^ At initial presentation, a substantial proportion of patients report a high prevalence of symptoms, such as, fatigue, dyspnea, and pain.^5^ These patients report clinically relevant worse fatigue compared to the general population^6^ and, even patients with lower‐risk disease have a poorer HRQoL profile than their peers from the general population.^7^ Measures used to assess HRQoL typically include various domains covering multidimensional aspects such as physical and social functioning as well as symptoms. However, there are also other type of measures that only focus on more specific aspects, such as symptom burden. In any case, as long as this type of information is obtained by patients themselves, we can refer to the more general term of patient‐reported outcomes (PROs).^8^

The importance of rigorously monitoring HRQoL in these patients has been emphasized in international guidelines.^2^ Likewise, HRQoL was selected as a relevant factor in a recently developed core MDS outcome set by experts in the field,^9^ and identified as one of the most relevant PROs both by patients with MDS and hematologists.^10^

Validated PRO measures are critical to facilitate clinical decision‐making, as they are devised to capture the direct perception of patients on the burden of disease and treatment and have been shown to provide unique information that cannot be captured via traditional clinical or biological markers.^11^ For example, patient‐reported fatigue in MDS cannot be merely explained by hemoglobin levels. Recent studies showed that MDS has an anemia‐independent impact on HRQoL,^12^ and some have explicitly reported a weak association between fatigue and anemia.^13^ Fatigue, as reported by patients themselves, has also been successfully incorporated into well‐established disease risk classifications to enhance their prognostic accuracy in higher‐risk MDS patients.^14^, ^15^, ^16^

To date, HRQoL in MDS research has been frequently assessed with non‐MDS‐specific measures,^17^ possibly limiting our understanding of the full breadth of problems experienced by these patients. Disease‐specific measures are more likely to capture key elements of HRQoL most relevant to the population being studied.^18^ Two PRO measures have been developed to be used with patients with MDS, that is, the Quality of Life‐E (QOL‐E)^19^ and the Quality of Life in Myelodysplasia Scale (QUALMS).^20^ A prior validation of the QUALMS has been reported^20^; however, this was largely based on data obtained in a North American cohort and only featured two administrations over 6 months. Moreover, data on the validity of its three subscales, that is, physical burden (QUALMS‐P), emotional burden (QUALMS‐E), and benefit findings (QUALMS‐BF), are scarce.

In an effort to raise quality standards of HRQoL assessment for patients with MDS, we integrated the QUALMS into a prospective non‐interventional European Registry study (i.e., MDS‐RIGHT Project) with the main goal of validating its three subscales.

## 2 PATIENTS AND METHODS

### 2.1 Patient population

The current study is part of the European Horizon 2020 MDS‐RIGHT Project “Providing the right care to the right patient with MyeloDysplastic Syndrome at the right time” (https://mds‐europe.eu/right) within the European LeukaemiaNet MDS (EUMDS) Registry. The EUMDS Registry (NCT 00600860) is a prospective, multicenter, non‐interventional study in patients with MDS from 16 European countries and Israel, which started in 2008.^21^ The sub study on QUALMS was approved by the EUMDS Steering committee. The QUALMS was integrated in the EUMDS Registry in January 2017 and has been applied in centers in the Netherlands, the United Kingdom, Israel, and Austria. It was administered at study entry (baseline) and then at 6, 12, 18, and 24 months. The EUMDS Registry was approved by the ethics committees of all participating centers and was performed in accordance with the Declaration of Helsinki. Written informed consent was obtained from all patients.

### 2.2 Initial QUALMS development and validation process

The QUALMS was developed by Abel G. and colleagues^22^ through the use of structured interviews with 32 MDS patients, caregivers, and clinicians. Subsequently, it was validated in an international cohort of 255 MDS patients.^20^ The QUALMS is a 38‐item measure, containing 33 items that are used for scoring and 5, individual “opt‐out” questions, which are not scored with the other items. It includes three subscales, namely, physical burden (QUALMS‐P, 14 items), emotional burden (QUALMS‐E, 11 items), and benefit finding (QUALMS‐BF, 3 items). A total score is calculated from the 33 core items, and the scores ranges from 0 to 100, with a higher score indicating better HRQoL outcomes. An overview of the questionnaire structure and item topics per domain is reported in the Table S1. This questionnaire is copyrighted by the Dana‐Farber Cancer Institute in Boston (USA). It has been translated into 42 languages, and licenses are free for use in academic studies. Registration procedures for using the QUALMS are available at: https://qualms.dana‐farber.org.

### 2.3 Statistical analyses

Baseline patient characteristics were reported by proportions or medians and interquartile ranges (IQR). Depending on the variable type, the Wilcoxon–Mann–Whitney test or the Fisher exact test were used to examine possible differences in socio‐demographic and clinical characteristics between patients with and without a completed QUALMS at study entry.

Descriptive statistics (i.e., mean, standard deviation [SD], median, minimum and maximum scores, skewness, and kurtosis values) were investigated for the three QUALMS subscales and the QUALMS Total (hereafter, scales). The presence of floor and ceiling effects at the scale level was also examined as this may negatively impact on sensitivity and responsiveness.^23^ For the purpose of this study, we used previously defined criteria suggesting that floor or ceiling effects are present if more than 15% of respondents obtain the lowest or highest possible score, respectively.^24^, ^25^

The internal consistency of the QUALMS scales was estimated using Cronbach's alpha,^26^ with a Cronbach's alpha coefficient ≥0.70 being considered acceptable.^27^ We performed a confirmatory factor analysis (CFA), using the weighted least squares estimator with adjustment for means and variances procedure, to examine the model fit for the underlying scale structure of the QUALMS. We used the Comparative Fit Index (CFI), the Tucker‐Lewis Index (TLI), and the Root Mean Squared Error of Approximation (RMSEA) to evaluate the goodness‐of‐fit of the model.^28^ CFI and TLI values above 0.95 indicate good fit, while values above 0.90 indicate acceptable fit. RMSEA values below 0.05 indicate good fit and values below 0.08 indicate acceptable fit.^29^ In addition, Spearman's rank correlation analyses were performed to examine the correlations between the QUALMS Total score with the three subscales.

Concurrent validity was assessed by performing Spearman's rank correlation analyses between the scales of the QUALMS and the EQ‐5D‐3L. We hypothesized that patients with higher scores on all scales of the QUALMS also had better outcomes (i.e., less severe or frequent problems) in the five dimensions of the EQ‐5D‐3L (mobility, self‐care, usual activities, pain, and anxiety/depression) and a higher score (better health status) in EQ‐VAS.

Known‐group comparisons were carried out to evaluate the discriminant validity of the QUALMS, using the Wilcoxon‐Mann–Whitney test to assess differences between patient subgroups. We compared the QUALMS scores in the following patient subgroups: MDS‐Comorbidity Index (CI)^30^ (low vs. intermediate/high), Karnofsky performance status (KPS) (< 90 vs. ≥ 90), anemia (anemic vs. non‐anemic patients) as defined by the WHO,^31^ sex (male vs. female), and red blood cell (RBC) transfusions within 1 year from completing the baseline QUALMS assessment (yes vs. no).

Responsiveness to change of the QUALMS scales was assessed by examining differences between baseline and follow‐up data for patients who reported an improvement in Hb levels (≥1.5 g/dL) from baseline (only for patients with a baseline Hb level < 11 g/dL).^32^, ^33^ For this analysis, we selected the follow‐up data of the QUALMS at which the first Hb improvement occurred. The level of statistical significance of all tests was set at α = 0.05. All analyses were performed with the R software version 3.6.0.

## 3 RESULTS

### 3.1 Patient characteristics

As of August 2020, the QUALMS was completed by 270 (87.6%) out of 308 MDS patients, who agreed to participate in the study from 17 centers across four countries (Austria [*N* = 61], Israel [*N* = 67], the Netherlands [*N* = 37], the United Kingdom [*N* = 105]). No statistically significant differences were observed in key sociodemographic and clinical factors, including age, sex, comorbidity, IPSS risk category, and having previously received RBC transfusions, between those who did not complete the QUALMS (*N* = 38) and those who did, considered in current analysis (*N* = 270) (data not shown).

Median age at study entry of the 270 patients analyzed was 74.0 years (IQR = 68.0–80.0), the majority of patients were male (67.4%) and had a low MDS‐CI score (60.3%). Further details are provided in Table 1.

**TABLE 1:** Patient characteristics at study entry

Variables	*N* = 270
Sex, N (%)	
Male	182 (67.4)
Female	88 (32.6)
Time since diagnosis, years	
Median (IQR)	0.5 (0.1–3.4)
Age at study entry, years	
Median (IQR)	74.0 (68.0–80.0)
MDS‐Comorbidity Index, N (%)	
Low	158 (60.3)
Intermediate	87 (33.2)
High	17 (6.5)
Missing	8 (.)
IPSS risk category, N (%)	
Low	103 (50.5)
Intermediate‐1	65 (31.9)
Intermediate‐2	26 (12.7)
High	10 (4.9)
Missing	66 (.)
IPSS‐revised risk category, N (%)	
Very Low	64 (34.2)
Low	58 (31.0)
Intermediate	32 (17.1)
High	16 (8.6)
Very High	17 (9.1)
Missing	83 (.)
Karnofsky performance status, N (%)	
≥90	98 (45.0)
<90	120 (55.0)
Missing	52 (.)
Received RBC transfusions, N (%)	
Yes	75 (29.3)
No	181 (70.7)
Missing	14 (.)
Hemoglobin level, g/dL	
Median (IQR)	10.0 (8.6–11.6)
WBC count, 10^9L	
Median (IQR)	4.4 (2.9–7.0)
Neutrophils, %	
Median (IQR)	51.0 (33.0–64.7)
Platelets, 10^9L	
Median (IQR)	129.0 (74.0–261.0)
Serum Ferritin level, ug/L	
Median (IQR)	345.0 (138.1–576.0)

Abbreviations: IQR, interquartile range; MDS, Myelodysplastic Syndromes; RBC, red blood cell; WBC, white blood cells.

### 3.2 Questionnaire characteristics and reliability of the QUALMS

Table 2 shows the descriptive statistics, displaying the score distribution for each QUALMS scale. Three out of four scales (i.e., QUALMS‐P, QUALMS‐E, and QUALMS Total) did not include the minimum score of 0 in their range. All scores had fairly symmetrical distribution with slight tendency towards higher values. The entire range of scores (0–100) was only observed for the QUALMS‐BF and all median scores ranged between 50 and 70 point. No floor and ceiling effects were observed at the scale level, as less than 15% of respondents achieved the lowest or highest possible scores in all three subscales and in the QUALMS Total.

**TABLE 2:** Distribution characteristics of the scales of the QUALMS

Scale	Mean	SD	Median	Min	Max	Skew	Kurtosis
QUALMS‐P	63.10	21.80	64.29	7.14	100.00	−0.19	−0.81
QUALMS‐E	69.71	19.59	70.45	4.55	100.00	−0.50	−0.09
QUALMS‐BF	50.38	25.58	50.00	0.00	100.00	−0.22	−0.47
QUALMS Total	66.22	16.31	68.18	15.15	96.97	−0.48	−0.18

*Note*: Higher QUALMS scores indicate better quality of life.

Abbreviations: QUALMS‐BF, benefit finding; QUALMS‐E, emotional burden; QUALMS‐P, physical burden; SD, standard deviation.

**FIGURE 1:**
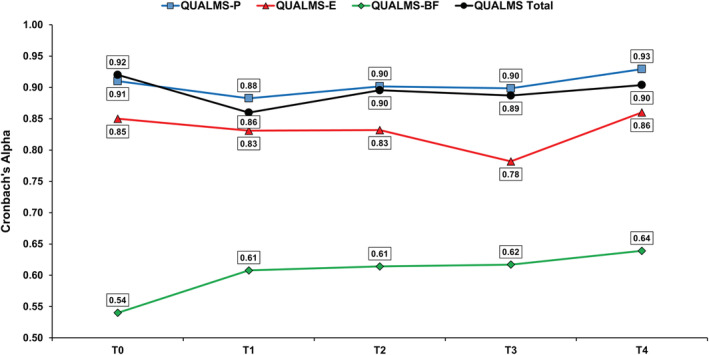
Cronbach's alpha values of the QUALMS over time. For each QUALMS scale, the figure represents the corresponding internal consistency at each assessment, that is, T0 = baseline, T1 = 6 months, T2 = 12 months, T3 = 18 months, T4 = 24 months. Connecting lines are drawn only for illustrative purposes. QUALMS‐BF, benefit finding; QUALMS‐E, emotional burden; QUALMS‐P, physical burden.

Figure 1 shows the Cronbach's alpha coefficients across serial assessments, that is, at 6 (*n* = 146, 55%), 12 (*n* = 99, 42%), 18 (*n* = 71, 34%), and 24 months (*n* = 36, 23%). The QUALMS‐P scale exhibited the highest coefficients across all timepoints, ranging from 0.88 to 0.93.

Results of the CFA showed support for the underlying scale structure of the QUALMS. All items of the QUALMS‐E exceeded the threshold of 0.40 (range = 0.56–0.73), while one item of the QUALMS‐P (range = 0.30–0.90) and one item of the QUALMS‐BF (range = 0.32–0.75) remained below the threshold of 0.40. Further, the fit indices CFI (0.93), TLI (0.93), and RMSEA (0.07) indicated acceptable model fit. In addition, strong positive Spearman correlation coefficients between the QUALMS Total and the QUALMS‐P (*r* = 0.92, *p* < 0.001) and the QUALMS‐E (*r* = 0.87, *p* < 0.001) were found. A weak negative correlation was observed between the QUALMS Total and the QUALMS‐BF (*r* = −0.15, *p* = 0.034).

### 3.3 Concurrent validity

As displayed in Table 3, the QUALMS‐P, the QUALMS‐E, and the QUALMS Total showed moderate negative correlations with the EQ‐5D‐3L scales (range *r*: −0.26 to −0.67; all *p*s < 0.001) and a moderate positive correlation with the EQ VAS (range *r*: 0.41 to 0.60; all *p*s < 0.001). The directions of these correlations were consistent with the conceptual assumption. The QUALMS‐BF, however, did not show a statistically significant correlation with any of the EQ‐5D‐3L scales and the EQ VAS (all *p*s > 0.05).

**TABLE 3:** Correlations between the scales of the QUALMS and the EQ‐5D‐3L questionnaire

	EQ‐5D Mobility	EQ‐5D Self‐care	EQ‐5D Usual activities	EQ‐5D Pain/Discomfort	EQ‐5D Anxiety/Depression	EQ‐VAS
QUALMS‐P	**−0.49**	**−0.47**	**−0.67**	**−0.45**	**−0.44**	**0.60**
QUALMS‐E	**−0.26**	**−0.29**	**−0.37**	**−0.40**	**−0.43**	**0.41**
QUALMS‐BF	0.00	−0.06	0.04	0.00	0.14	−0.07
QUALMS Total	**−0.40**	**−0.41**	**−0.56**	**−0.46**	**−0.49**	**0.57**

*Note*: Higher QUALMS scores and higher EQ VAS scores indicate better outcomes, while higher scores on the EQ‐5D‐3L subscales indicate worse outcomes (i.e., more severe or frequent problems). Values in bold indicate statistically significant correlation coefficients (*p* < 0.001).

Abbreviations: QUALMS‐BF, benefit finding; QUALMS‐E, emotional burden; QUALMS‐P, physical burden; VAS, visual analogue scale.

### 3.4 Discriminant validity and responsiveness to change

Results of the known‐group comparisons are shown in Table 4. Patients with a low MDS‐CI score, reported significantly better scores for QUALMS‐P (*p* = 0.006), QUALMS‐E (*p* = 0.014), and the QUALMS Total (*p* = 0.006) than patients with an intermediate or high MDS‐CI score. Compared to patients with a lower Karnofsky performance status (< 90), patients with a higher Karnofsky performance status (≥ 90) demonstrated significantly better scores for QUALMS‐P (*p* < 0.001), QUALMS‐E (*p* = 0.001), and the QUALMS Total (*p* < 0.001). Anemic patients, compared to non‐anemic patients, reported significantly worse scores for QUALMS‐P (*p* < 0.001), QUALMS‐E (*p* = 0.010), and the QUALMS Total (*p* = 0.001). Mean and (SD) of Hb levels were 9.5 g/dL (1.8) and 13.7 g/dL (1.1), for anemic and non‐anemic patients, respectively. Transfusion‐dependent patients, compared to those who were not, reported significantly worse scores for QUALMS‐P (*p* < 0.001), QUALMS‐E (*p* = 0.002), and the QUALMS Total (*p* < 0.001). However, mean scores of the QUALMS‐BF did not go in the expected direction by performance status score, anemia or transfusion dependency.

**TABLE 4:** Differences in the scales of the QUALMS by the MDS‐CI, Karnofsky performance status, Anemia, and RBC transfusions

	MDS‐CI					Karnofsky performance status					Anemia^a^					RBC transfusions^b^				
	Low *N* = 143		Int./High *N* = 94		*p*	< 90 *N* = 110		≥ 90 *N* = 91		*p*	Anemic patients *N* = 218		Non‐Anemic patients *N* = 35		*p*	No *N* = 166		Yes *N* = 68		*p*
QUALMS Scale	Mean	SD	Mean	SD		Mean	SD	Mean	SD		Mean	SD	Mean	SD		Mean	SD	Mean	SD	
QUALMS‐P	66.6	21.3	58.6	21.9	0.006	54.6	19.4	75.8	17.9	<0.001	60.5	21.8	77.7	16.5	<0.001	68.8	20.4	49.6	18.4	<0.001
QUALMS‐E	72.8	17.7	65.8	21.3	0.014	67.0	18.7	75.3	19.3	0.001	68.1	19.4	78.5	17.9	0.010	72.2	19.0	63.5	19.5	0.002
QUALMS‐BF	50.8	26.5	48.8	24.3	0.483	50.9	23.2	49.4	29.7	0.829	51.3	25.0	43.8	29.9	0.243	47.9	25.9	57.4	21.0	0.014
QUALMS Total	69.1	14.8	62.2	17.6	0.006	61.8	15.0	74.2	14.5	<0.001	64.6	16.4	76.3	12.7	0.001	69.8	15.3	57.6	15.1	<0.001

Abbreviations: Int., intermediate; MDS‐CI, MDS‐comorbidity index; QUALMS‐BF, benefit finding; QUALMS‐E, emotional burden; RBC, red blood cell; QUALMS‐P, physical burden; SD, standard deviation.

^a^ According to the definition of the World Health Organization, female patients with hemoglobin (Hb) levels <12.0 g/dL and male patients with Hb levels <13.0 g/dL were classified as being anemic. Mean and (SD) of Hb levels were 9.5 g/dL (1.8) and 13.7 g/dL (1.1), for anemic and non‐anemic patients, respectively.

^b^ Denotes whether the patient has received any red blood cell transfusions within 1 year from the baseline QUALMS assessment.

**FIGURE 2:**
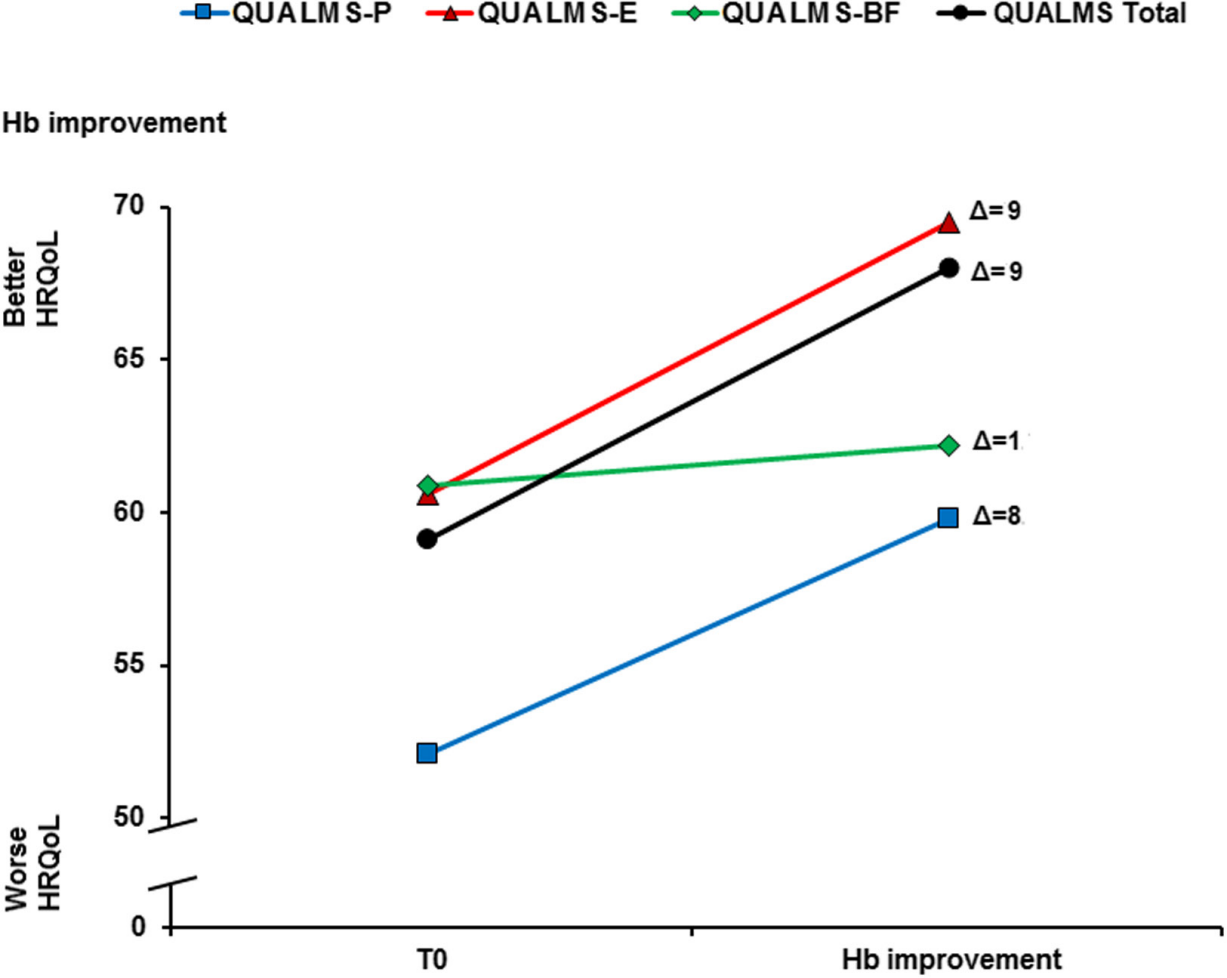
Responsiveness to change of the QUALMS by hemoglobin improvements. Figure shows the responsiveness to change of the QUALMS by meaningful improvement in hemoglobin values (≥1.5 g/dL) from baseline (only for patients with a baseline Hb level < 11 g/dL) (*n* = 30). Hb, hemoglobin; QUALMS‐BF, benefit finding; QUALMS‐E, emotional burden; QUALMS‐P, physical burden.

Responsiveness to change analysis indicated that patients with a Hb level of <11 g/dL at study entry, who subsequently showed an improvement in their Hb level (≥1.5 g/dL), also reported mean score changes (Δ) in the expected direction for the QUALMS Total (Δ = 9), and the subscales: QUALMS‐E (Δ = 9), QUALMS‐P (Δ = 8), QUALMS‐BF (Δ = 1) (Figure 2).

## 4 DISCUSSION

Our data provide novel information on the validity of the QUALMS which broadly support its use in patients with MDS. Factor analysis confirmed the hypothesized structure of the measure and fit indices were high; also, no floor and ceiling effects were observed. Compared to previous validation steps of the QUALMS,^20^ the current analysis provides more extensive information on the psychometric performance of its three subscales and additional data on responsiveness to change of this measure, which was initially only shown for patients who experienced infection and hospitalization. Taken together with prior validation efforts,^20^ the QUALMS has now been tested in two independent cohorts including overall more than 500 patients enrolled across 22 centers in seven countries (Austria, Canada, the Netherlands, Israel, Italy, the United Kingdom and the USA).

In particular, our data indicate the high performance of the QUALMS‐P, which captures specific physical health related aspects as well as fatigue and other key symptoms. Indeed, our discriminant validity analysis indicated large mean score differences (in the expected direction) among different clinical conditions highly relevant for the MDS population, including anemia and RBC transfusions. Also, reliability of the QUALMS‐P was good as indicated by a Cronbach's α of ≥0.90 across 4 of out 5 consecutive assessments. This is an important finding as physical function is a key PRO domain frequently associated with survival outcomes in cancer patients,^11^ and has been also recently included by the US FDA in the core PROs recommended for use in clinical trials.^34^

Inspection of the psychometric performance of the QUALMS‐E was also in the expected direction and overall good, similarly to what was observed in the initial validation steps.^20^ However, the clinical value of QUALMS‐BF remains to be elucidated in future works. Indeed, this scale may be less relevant in the context of comparative studies assessing drug efficacy, while it may be of further interest in other research settings. For example, benefit‐finding is an important aspect to consider in psychosocial research^35^ and may also depend on the specific timing of its assessment, with recent studies indicating that prevalence of benefit‐finding may be lower during the earlier years after diagnosis in some cancer populations.^36^ Hence, it would be interesting to include the QUALMS‐BF in future psychosocial research studies of patients with MDS, to better understand how benefit finding relates to disease and patient characteristics. This is an unexplored area of research for this cancer population.

While valuable advances have been made in recent years in MDS research, these have been mainly confined to biological and clinical research. The wealth of information currently available on the biology of the disease and of its clinical evolution, stands in sharp contrast with the scarcity of robust HRQoL data, for example, with regard to the patient‐relevant impact of different MDS therapies. While several reasons may account for the lack of more substantial efforts in HRQoL research in MDS, one reason is possibly the paucity of internationally validated disease‐specific PRO measures.

A recent systematic review on the most frequently used PRO measures in MDS research,^17^ found that the large majority of studies published in this area have used generic (i.e., EQ‐5D)^37^ or cancer‐generic questionnaires (i.e., EORTC QLQ‐C30).^38^ While both measures have greatly helped in providing key data from the patient's standpoint, these questionnaires were not specifically developed for patients with MDS and thereby may have not thoroughly captured specific aspects associated with the wellbeing of this patient population. Therefore, the availability of an MDS‐specific measure may contribute to refine the HRQoL assessment in this cancer population. Recent qualitative work in patients with lower‐risk MDS has indicated that the QUALMS has strong face and content validity,^39^ thereby lending further credibility to its clinical value in the context of MDS.

The QUALMS is already used in a large US‐based registry^40^ and our findings, obtained in European and Israeli patients, may support its implementation in clinical research in other countries. Future clinical trials in MDS should consider HRQoL endpoints to ensure treatment goals are meaningful to patients.^41^ Hence, the availability of an internationally validated MDS‐specific measure may increase accuracy of HRQoL aspects that matter the most to these patients. A recent study challenged the assumption that RBC transfusions to treat symptomatic anemia can improve HRQoL in all MDS patients. Using the QUALMS, investigators^42^ found that only about one‐third of patients experienced a clinically significant increase in the QUALMS Total after transfusion (35%), about half experienced no change (46%) and 19% experienced a decrease in HRQoL. Of note, in this study^42^ clinical significance was defined as a 5‐point change in the QUALMS Total score, although a more conservative approach would define the clinical significance of this scale as difference of 7.6 points, as reported in the prior validation.^20^ In the current study, for the QUALMS‐P, a distribution‐based method would argue that a clinically meaningful difference would be a half standard deviation, which would correspond to 9 points.

Implementation of the QUALMS, or of some of its scales, may possibly be considered in routine clinical practice to help clinicians better understand burden of disease and therapy from each individual patient's unique viewpoint. There is convincing evidence that regular PRO monitoring in clinical practice may have a number of valuable clinical implications.^43^ However, empirical evidence in the MDS arena is scant and future studies could examine how the QUALMS could provide valuable information in routine MDS practice.

Our study has limitations. Responsiveness to change analysis was limited to the evaluation of improvement in Hb levels. Therefore, it will be important to obtain in future studies further information on the performance of the QUALMS across various MDS therapies, including data on QUALMS scores changes in patients who become transfusion independent. Also, further work is needed to identify scale‐specific thresholds for determining clinical significance of results beyond the one that is suggested above for the QUALMS‐P scale. A strength of this study is the involvement of several centers across different countries, which lends further credit to generalizability of our findings.

In conclusion, our results support the validity of the QUALMS and provides novel information on the psychometric performance of its three subscales. This questionnaire may help clinicians to harness the patients voice both in clinical research and practice.

## AUTHOR CONTRIBUTIONS

**Fabio Efficace:** Conceptualization (lead); investigation (equal); methodology (equal); supervision (lead); writing – original draft (lead); writing – review and editing (equal). **Karin Anne Koinig:** Conceptualization (equal); investigation (equal); methodology (equal); writing – original draft (equal); writing – review and editing (equal). **Francesco Cottone:** Conceptualization (equal); formal analysis (equal); investigation (equal); methodology (equal); writing – original draft (equal); writing – review and editing (equal). **David Bowen:** Investigation (equal); methodology (equal); writing – review and editing (equal). **Moshe Mittelman:** Investigation (equal); methodology (equal); writing – review and editing (equal). **Kathrin Sommer:** Conceptualization (equal); formal analysis (equal); investigation (equal); methodology (equal); writing – review and editing (equal). **Saskia Langemeijer:** Investigation (equal); methodology (equal); writing – review and editing (equal). **Dominic Culligan:** Investigation (equal); methodology (equal); writing – review and editing (equal). **Kalman Filanovsky:** Investigation (equal); methodology (equal); writing – review and editing (equal). **Michael Storck:** Investigation (equal); methodology (equal); writing – review and editing (equal). **Alexandra Smith:** Investigation (equal); methodology (equal); writing – review and editing (equal). **Corine van Marrewijk:** Investigation (equal); methodology (equal); writing – review and editing (equal). **Martin Dugas:** Investigation (equal); methodology (equal); writing – review and editing (equal). **Igor Stojkov:** Investigation (equal); methodology (equal); writing – review and editing (equal). **Uwe Siebert:** Investigation (equal); methodology (equal); writing – review and editing (equal). **Theo De Witte:** Investigation (equal); methodology (equal); writing – review and editing (equal). **Reinhard Stauder:** Conceptualization (lead); investigation (equal); methodology (equal); supervision (lead); writing – original draft (equal); writing – review and editing (equal).

## FUNDING INFORMATION

This work is part of the MDS‐RIGHT activities, which has received funding from the European Union's Horizon 2020 research and innovation programme under grant agreement No 634789—“Providing the right care to the right patient with MyeloDysplastic Syndrome at the right time.” This study was supported by Senioren‐Krebshilfe, www.senioren‐krebshilfe.at (KAK, RS). The EUMDS Registry is supported by educational grants from Novartis Pharmacy B.V. Oncology Europe, Amgen Limited, Celgene International, Janssen Pharmaceutica, and Takeda Pharmaceuticals International.

## CONFLICT OF INTEREST

Fabio Efficace: personal fees from Amgen, Abbvie, Janssen, and Novartis. Research support (to Institution) from Abbvie, Amgen, Novartis, all unrelated to this work. Corine van Marrewijk: project manager of the EUMDS Registry, is funded from the EUMDS (educational grants from Novartis Pharmacy B.V. Oncology Europe, Amgen Limited, Celgene International, Janssen Pharmaceutica, and Takeda Pharmaceuticals International) and MDS‐RIGHT (grant from EU's Horizon 2020 programme) project budgets. Moshe Mittelman: research grant: Janssen, Roche, Novartis, Medison/Amgen, Celgene/BMS, Abbvie, Gilead. Clinical trials sponsored by: Novartis, Takeda, Fibrogen, Celgene/BMS, Geron. Advisory board: Onconova, Novartis, Takeda, Silence, Astellas. Consulting fees: MDS HUB. Speakers' bureau: Celgene/BMS, Novartis, Media Digital. Reinhard Stauder: Celgene, BMS, Novartis (Advisory board); Celgene/BMS, Novartis (Honoraria); Celgene and BMS (Research funding), all unrelated to this work. Theo de Witte: Research Funding: Amgen, Celgene, Janssen, Novartis, Takeda.

## ETHICAL APPROVAL

The EUMDS Registry was approved by the ethics committees of all participating centers and was performed in accordance with the Declaration of Helsinki. Written informed consent was obtained from all patients.

## Supporting information


Table S1

## Data Availability

Data may be available upon request.
