# Supplementary material for: Raising the standards of patient‐centered outcomes research in myelodysplastic syndromes: Clinical utility and validation of the subscales of the QUALMS from the MDS‐RIGHT project
Source: Cancer Med. 2022 Dec 19;12(6):7529–39. doi: 10.1002/cam4.5487 (PMC10067097; doi:10.1002/cam4.5487)
Supplement: Supplementary file 1 — Table S1 [file CAM4-12-7529-s001.docx]

**Supplementary Table 1.** Overview of patient-reported outcome domains covered by the QUALMS questionnaire* (38 questions with 33 included in the overall score and 5 optional/single response)

| **Category** | **Scale abbreviation** | **n. of items**  (cross-hatched are in overall score**)** | **Issues addressed** |
| --- | --- | --- | --- |
| Physical burden | QUALMS-P | **14** | Too tired for prior responsibilities  Low energy change schedule  Weak  Unable participate in activities  Take into account might be fatigued  Worry about becoming burden  Felt hopelessness  Change in bowels  Shortness of breath  Change long-term plans due to health  Trouble concentrating  Life organized around medical  Nauseated  Energy for routine tasks |
| Emotional Burden | QUALMS-E | **11** | Could not do anything about disease  Disease unpredictable  Lack of concrete answers  No clear information  Afraid of dying  Difficulty explaining MDS to others  Worry progressing/leukemia  Anxious about tests or lab results  Angry about diagnosis  Worried infection  Limited emotional support available |
| Benefit finding | QUALMS-BF | **3** | Grateful for tomorrow  Get quality information  Gratitude when prior took for granted |
| Part of overall score, not in sub-scale | N/A | **5** | Family relationships strained  Bruising  Avoid crowds  Worried bleeding  Concerned financial burden |
| Single response/optional (not in overall score or sub-scale) | N/A | **5** | Fear of losing job  Too tired to drive  Afraid to have sex due to counts Worry MDS treatment stop working Too tired to take care of family |

* Abel G, et al. Haematologica. 2016;101(6):781-788.

Abbreviations: QUALMS-P = physical burden; QUALMS-E = emotional burden; QUALMS-BF = benefit finding
